# Leveraging Generative Artificial Intelligence to Improve Motivation and Retrieval in Higher Education Learners

**DOI:** 10.2196/59210

**Published:** 2025-03-11

**Authors:** Noahlana Monzon, Franklin Alan Hays

**Affiliations:** 1 Department of Nutritional Sciences, University of Oklahoma Health Sciences, 1200 N Stonewall Ave, 3064 Allied Health Building, Oklahoma City, OK, 73117, United States, 1 405 2718001 ext 41182

**Keywords:** educational technology, retrieval practice, flipped classroom, cognitive engagement, personalized learning, generative artificial intelligence, higher education, university education, learners, instructors, curriculum structure, learning, technologies, innovation, academic misconduct, gamification, self-directed, socio-economic disparities, interactive approach, medical education, chatGPT, machine learning, AI, large language models

## Abstract

Generative artificial intelligence (GenAI) presents novel approaches to enhance motivation, curriculum structure and development, and learning and retrieval processes for both learners and instructors. Though a focus for this emerging technology is academic misconduct, we sought to leverage GenAI in curriculum structure to facilitate educational outcomes. For instructors, GenAI offers new opportunities in course design and management while reducing time requirements to evaluate outcomes and personalizing learner feedback. These include innovative instructional designs such as flipped classrooms and gamification, enriching teaching methodologies with focused and interactive approaches, and team-based exercise development among others. For learners, GenAI offers unprecedented self-directed learning opportunities, improved cognitive engagement, and effective retrieval practices, leading to enhanced autonomy, motivation, and knowledge retention. Though empowering, this evolving landscape has integration challenges and ethical considerations, including accuracy, technological evolution, loss of learner’s voice, and socioeconomic disparities. Our experience demonstrates that the responsible application of GenAI’s in educational settings will revolutionize learning practices, making education more accessible and tailored, producing positive motivational outcomes for both learners and instructors. Thus, we argue that leveraging GenAI in educational settings will improve outcomes with implications extending from primary through higher and continuing education paradigms.

## Introduction

**Figure 1.**
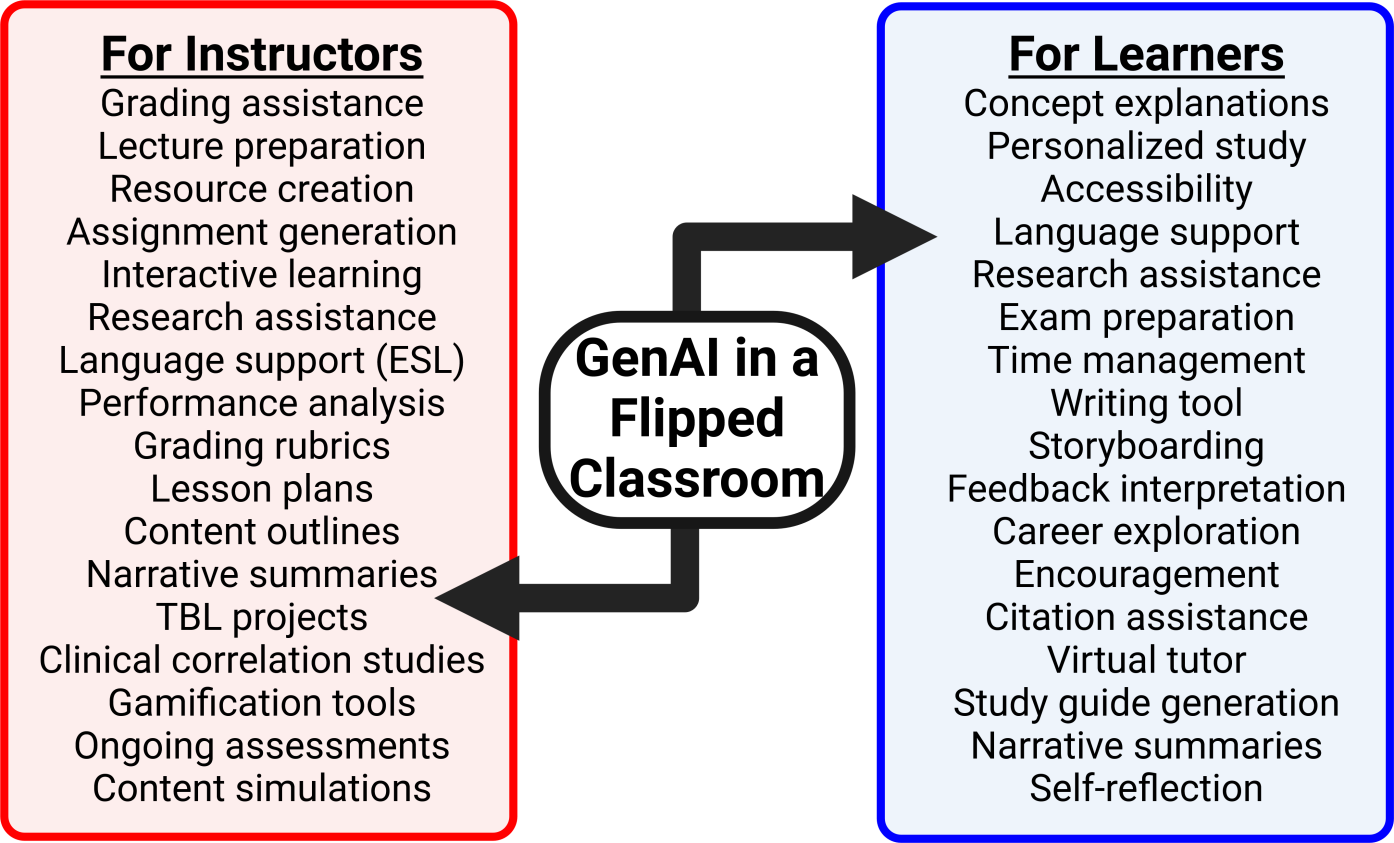
Generative artificial intelligence application in a flipped classroom model enhances both learner (blue) and instructor (red) experiences. Shown are examples of generative artificial intelligence benefit and impact within respective domains. Bidirectional arrows indicate reciprocal enhancement of generative artificial intelligence applications, demonstrating improvements in instructor-driven activities inherently enrich learner experience, thereby reinforcing a flipped classroom learning environment. GenAI: generative artificial intelligence.

Generative artificial intelligence (GenAI) is impacting educational spaces in ways that few technologies have since the personal computer and calculator [1-3]. Though GenAI is not a new concept, its inroads into education and pedagogy started in earnest following the release of “ChatGPT” (November 30, 2022). We observed learners using ChatGPT within weeks of its release. GenAI continues to rapidly evolve with new “GPTs,” models, websites, application programming interfaces, and GenAI-enabled hardware [3-6]. GenAI is now “mainstream” with low activation barriers for use. This new reality is sending shockwaves through educational institutions and districts, including higher and clinical education. Learners, instructors, and administration work to understand and define implications while either leveraging or obstructing GenAI implementation. Indeed, banning and blocking GenAI use in some educational settings remains with no clear consensus on what role GenAI can, or should, play going forward. With this rapidly evolving space, it’s challenging to differentiate inflated expectations and hype from productivity and enlightenment, to borrow from the Gartner Hype Cycle. One could argue that the GenAI “peak of inflated expectations” has yet to be reached. However, the new reality in clinical and higher education is one where GenAI will play a role going forward, both within classrooms and clinical practice [3,6-8]. What that looks like remains variable and will change depending on the knowledge of those involved, personal perspectives on GenAI, learner and programmatic needs, and accreditation standards and expectations. Our immediate approach was to embrace GenAI, like ChatGPT and other tools as they have come online (eg, Claude, Bard, and CoPilot), as a new tool to facilitate learning, retrieval, and motivation in a higher education, clinically focused, instructional environment. The rationale is that modern GenAI can generate diverse outputs (eg, text, images, videos, and language) derived from data-centric training sets [9] using narrative style prompts or data inputs (eg, content outlines, documents, PDFs, and images). Thus, there is a pragmatic reality that new GenAI tools have a low activation barrier for use while being capable of generating high-quality output focused on user needs. It’s evident that modern GenAIs ability to generate extensive, coherent, responses is fundamental to increasing engagement, communication, and motivation in educational settings. Though not seamless, especially when considering potential for malfeasance or hallucinations [10], GenAI can integrate throughout curricula to reduce overhead and improve outcomes (Figure 1). This integration fosters environments that inspire and empower learners, promote motivation and collaboration, and facilitates the creation of dynamic and individualized curricula.

The objective of this viewpoint is to advance a positive perspective on leveraging GenAI tools in modern medical education environments while presenting examples and methods tested in our hands since ChatGPT’s initial release. This viewpoint is presented from both learner (Mrs Monzon) and instructor (Dr Hays) perspectives as, in our experience, both offer unique opportunities on GenAI use. Learners are focused on knowledge acquisition and retrieval from an individualized perspective. Not all learners have the same motivation, hidden curriculum, previous knowledge, and experience, or ability to learn and retain learning objectives defined by instructors. Likewise, instructors are unable to individualize curricula across multiple learners or sections while ensuring productive exposure to core learning objectives defined by accreditation and program standards. It’s a conundrum of modern higher education, learners seeking individualized instruction amidst information overload while instructors are bandwidth-limited and hamstrung by program and accreditation demands. GenAI tools will directly impact this reality in a positive manner and empower both learners and instructors. The current challenge is what does that look like? How can GenAI be integrated into learning environments to facilitate learning and retrieval, drive motivation, and improve outcomes while avoiding pitfalls such as loss of voice, data ownership and use, academic misconduct or malfeasance, and incorrect information? This future must balance innovation and GenAI integration with established guidelines, integrity and safety guardrails, and equity. By presenting a nuanced perspective of the interplay between GenAI and learning theories from both learner and instructor perspectives, this viewpoint intends to inform GenAI integration that is inclusive, forward-thinking, and collaborative while not ignoring tangible GenAI benefits for all stakeholders in the learning ecosystem. Integration should not overshadow essential human elements of teaching and learning but rather complement and enhance both, thereby creating a dynamic and inclusive educational environment that is responsive. Finally, we argue that GenAI should not be ignored but embraced. It’s imperative that learners are exposed to new technologies that will increasingly impact workforce dynamics going forward. Instructors are innovators and our learners are digital natives surrounded by AI technologies. We implemented and evolved methods described in this viewpoint within graduate (PhD and MS), clinical (dietetics and RD), and undergraduate curricula. Leveraging GenAI in courses does require initial effort, yet subsequent improvements in effort needed, instructional quality, and learner feedback justify the initial cost. GenAI has proven, in our hands, to positively impact every pedagogical niche. It should be noted that we acknowledge significant ethical concerns regarding GenAI use in educational settings. This has been covered extensively elsewhere [10,11] and the current viewpoint starts with the perspective that GenAI can, and should, play a role in educational settings.

## Learner Perspective

**Figure 2.**
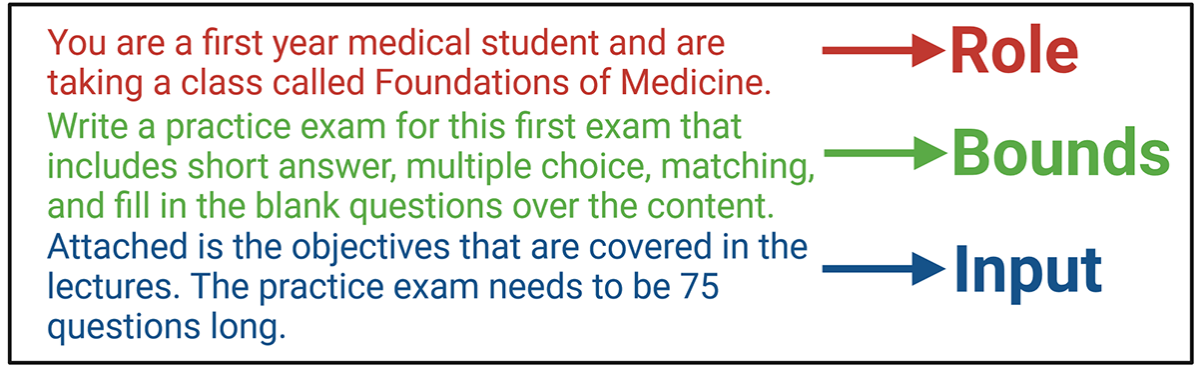
Prompt design. General overview for developing prompts with clearly defined role (red), bounds (green), and input (blue). Each component is essential for effective generative artificial intelligence use toward minimizing refinement while helping ensure output is optimized toward specific needs.

Technology is a powerful means to facilitate collaboration between learners and instructors. Learning management systems (eg, Canvas or D2L) are an example of this point, leveraging technology to facilitate learning and retention. In this sense, bringing GenAI into classroom settings is an evolutionary step with clear emerging data that it enhances learner engagement, motivation, and personalized learning in a self-directed manner. The pragmatic meaning is that interactive collaboration can be extended from instructor-learner or learner-learner to include learner-GenAI where the scope and implementation of learner-GenAI interactions is defined by tools being used, prompt design (Figure 2), and personalized needs (Figures1  3). This approach fosters learner motivation as a key driver for positive outcomes [12]. In addition, learning is more effective when it’s relevant, engaging, and contextualized to real-life scenarios (eg, team-based learning or clinical applications) in accordance with adult learning theory. Cognitive load is reached when germane, intrinsic, and extraneous factors become unmanageable [13]. Incorporating GenAI into the educational framework can simplify the intrinsic load, reduce the extraneous load and in turn maximize the germane load. This is consistent with our observations using GenAI to foster collaborative interactions in clinical courses. To maintain learner motivation, one must account for both intrinsic and extrinsic factors [13]. Intrinsic factors include self-efficacy, self-determination, curiosity, cognitive engagement, emotional well-being, professional well-being, and innate interest in the material presented. Extrinsic factors include pedagogical approach, peer interactions, assessment methods, learning environment, curriculum design, and quality and scope of feedback (both peers and instructors). A unique aspect to the learner-GenAI interaction is that it impacts both intrinsic and extrinsic motivational elements for learners. For instance, GenAI can be implemented as a personal tutor or study partner that encourages conversations and positive feedback in a low activation barrier environment (eg, compared with instructor office hours). Engaging GenAI “chatbots” like ChatGPT can also be conversational for learners, like interacting with a human counterpart (see “Current Limitations and Future Hurdles” section below). Thus, leveraging the learner-GenAI interaction provides agency to learners which increases autonomy and motivation [14].

**Figure 3.**
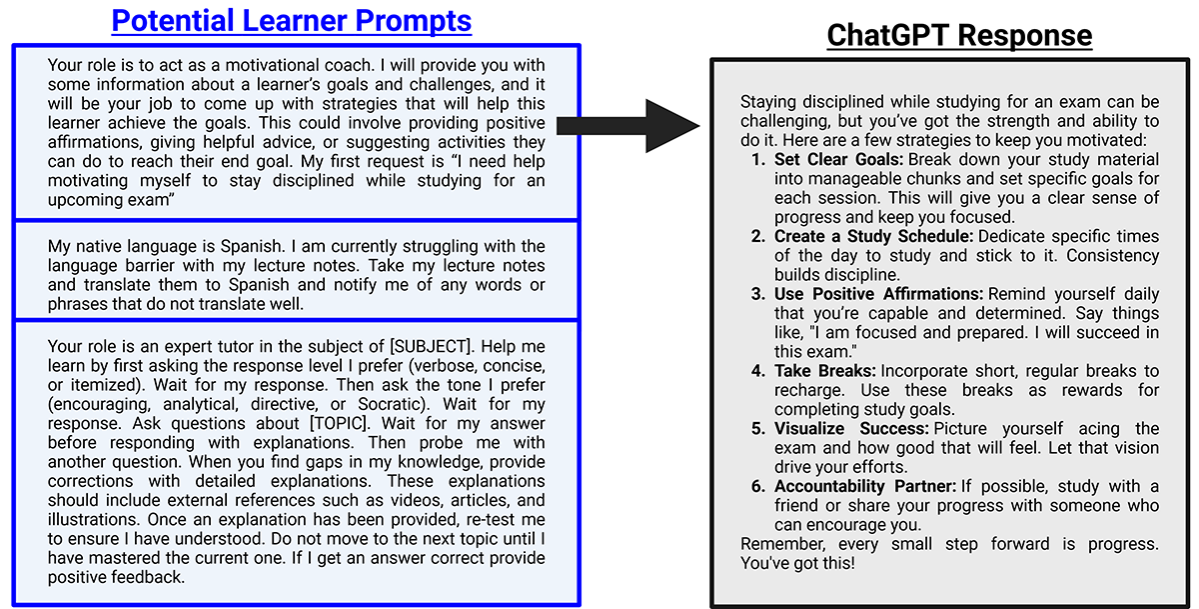
Example learner prompts for leveraging generatice artificial intelligence. Three example learner input prompts are shown (blue box) with representative ChatGPT (accessed on December 10, 2024) output (black box) for the top prompt (black arrow). In these instances, the resultant output can change significantly with minor changes in the input prompt provided. This is also true when using the same input prompt across different generatice artificial intelligence tools. Thus, specificity and clear instructions are key to effect desired output.

Beyond motivation, retrieval practice is an essential component for learner ownership over new material and, for example, applying knowledge in a clinical setting where integration and rapid access is often required [13]. Indeed, learned information is rapidly forgotten without reinforcement [15]. This is a core consideration of the “Desirable Difficulties” theory by Bjork and Bjork [15] suggesting that one should introduce challenges (eg, spacing or testing effects and varied practice) to enhance long-term memory retention of new information. Thus, retrieval practice requires active effort by learners to bolster information recall. This active engagement promotes deeper processing and understanding to facilitate ownership. A common example of retrieval practice in medical learner training is leveraging the Socratic method in clinical rounds, case discussions, simulations, journal clubs, team-based learning, and even mortality and morbidity conferences. In these scenarios, clinicians are pushed to understand, integrate, and verbalize knowledge under immediate critique and assessment. This moves beyond simple passive recall or reading to test true understanding and identify areas where learners assume ownership of knowledge but fail accurate retrieval or application [16]. In simplistic terms, retrieval practice is a common element to most curricula through formative (common in direct clinical training) and summative (common in formalized classroom instruction) assessments. Incorporating learner-GenAI methods into the curriculum provides a dynamic, ongoing, personalized, and iterative method to facilitate retrieval practice for learners outside of formal, instructor-based, course design. The learner-GenAI axis is instructor-independent in this instance. GenAI can generate adaptive quizzes and assessments while customizing difficulty level and content based on learner proficiency (eg, Figure 3). As learners progress and improve in retrieval practice, GenAI can dynamically adjust question complexity, ensuring continued adaptive learning. These tools analyze users learning patterns, preferences, performance data, and needs to personalize content. In this instance, GenAI recommends specific retrieval or practice exercises and intervals to drive memory consolidation. Learner-GenAI natural language interactions can efficiently manage spaced repetition schedules based upon individual learning patterns and needs to adapt timing and frequency of review sessions, ensuring learners revisit information at optimal intervals for memory retention. Finally, it’s important for instructors to consider that learners do not enter courses on equitable footing in knowing how to access, use, and leverage GenAI tools. Initial training with pragmatic examples, discussion of prompt engineering, setting up accounts if needed, and reviewing available tools and associated strengths and weaknesses is strongly advised for courses that allow GenAI use.

## Instructor Perspective

Instructors, faculty, and programs within higher education and clinical training settings are primary determinants of motivational factors for learners [12-14]. These include accreditation and departmental oversight (meaning “static” curriculum), designing and structuring assessment, determining feedback mechanisms, managing learning environment, defining expectations, and even implementing recognition and reward systems for positive outcomes or performance. Thus, in our experience, learner motivation is impacted in significant ways before the first day of class. With this backdrop in place, what role can GenAI play in higher education and clinical training from the instructors’ perspective? This question has interesting parallels to the “great calculator debate.” These include questions of access and equity, learners using these tools outside class regardless of policies set by schools and instructors, learners not gaining essential skills, or knowledge, through their use, modifications required for existing curricula with a major shift away from algorithms and “rules” toward meaning, concepts, and applications, and ability to trust accuracy of answers produced from novel technologies. Yet, even with the rapidly evolving current landscape surrounding GenAI, we posit that GenAI has significant benefits for instructors. Thus, the instructors’ role in harnessing GenAI as an educational tool is multifaceted and includes instructional design, creating dynamic learning content, and even streamlining administrative tasks (Figures1  4), all of which are predicated on training and learning about a rapidly evolving field with new tools appearing almost daily.

**Figure 4.**
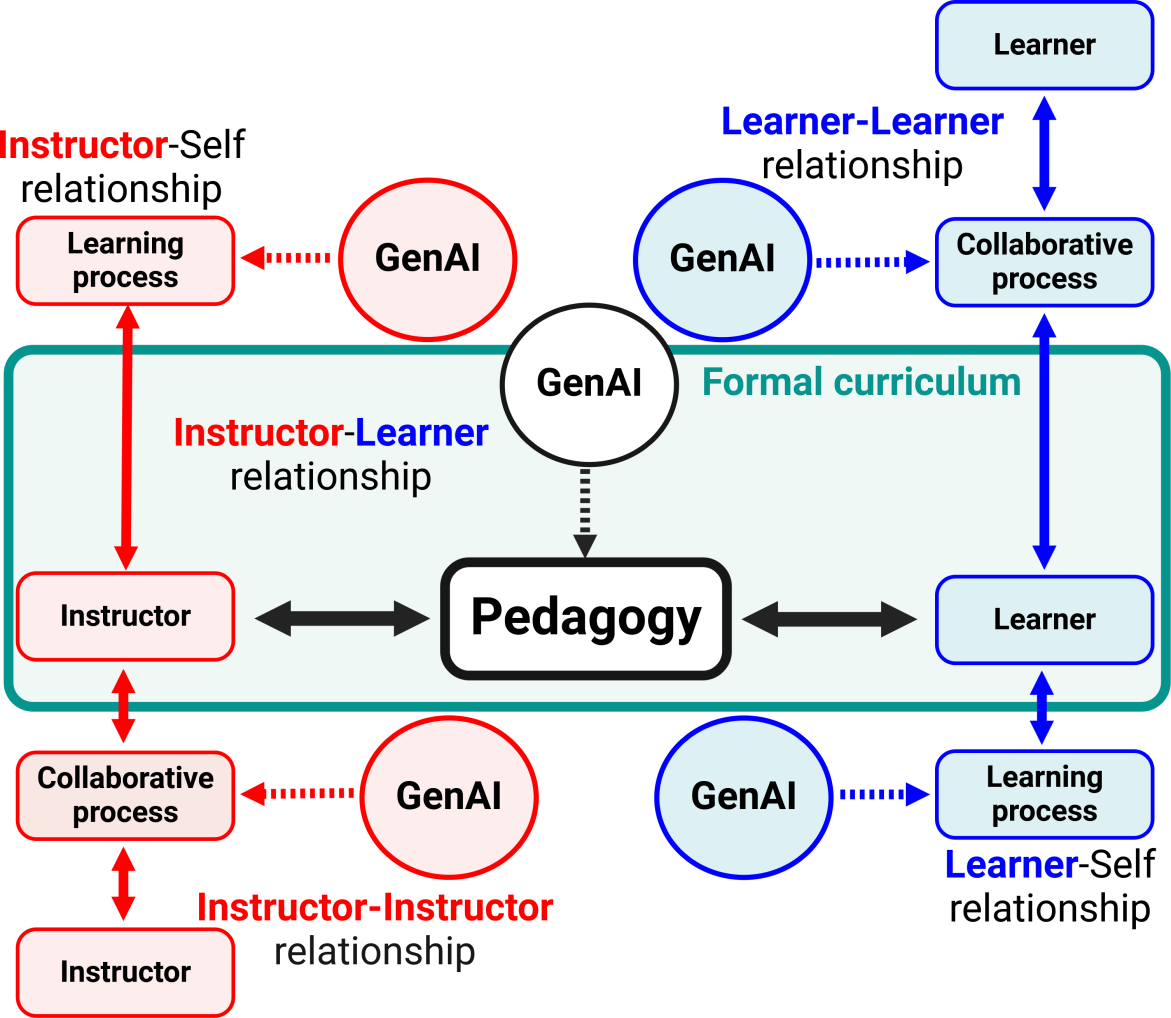
Role of generative artificial intelligence in educational relationships and processes. Generative artificial intelligence intersects with and supports relational dynamics between instructors (red) and learners (blue). Four primary interactions are shown, instructor-self, instructor-learner, learner-self, and learner-learner, where generative artificial intelligence serves as a pivotal tool for enhancing learning processes. Generative artificial intelligence’s contribution to the pedagogical framework is central, mediating and enriching explicit curriculum delivery and assimilation. Bidirectional arrows between actors and generative artificial intelligence signify a feedback loop allowing for continuous improvement of educational strategies. It underscores generative artificial intelligence’s potential to facilitate collaborative processes as well as promoting self-directed learning and peer-to-peer engagement. GenAI: generative artificial intelligence.

GenAI integration by instructors can lower activation barriers to create dynamic, engaging, personalized, and efficient learning environments that optimize learner outcomes (inclusive of motivation and retrieval). We approach this using a flipped classroom (Figure 1), content gamification, streamlined workflows, team-based learning, knowledge gap analysis, and consistent feedback using “exit tickets,” all facilitated using GenAI tools. The concept of classroom “flipping” has gained attention in recent years as an approach to instructional delivery. Flipping involves inverting traditional curriculum structure with learners acquiring, or at least engaging, new content outside of structured class time and using active learning methods during class to reinforce, expand upon, and use retrieval practice to reinforce and learn content [17]. All of which is instructor-guided as part of the instructor-learner core axis (Figure 4) [18]. If done well, this approach allows instructors to focus on providing targeted and personalized feedback, requires higher-order critical assembly and thinking skills, and facilitates meaningful discussion or reinforcement during class time for learners. Core tenants of this approach are engaging motivation for learners and expanded retrieval practice outside of high-stress graded assessments like quizzes or exams. Though our experience with the above approach involves class sizes ranging from 5‐40 learners, others have successfully implemented flipped classroom methods with more than 200‐300 learners [19]. Great examples of this dynamic approach are “metabolic melodies” in which the instructor, Dr Kevin Ahern from Oregon State University, uses complex biochemistry content to generate songs set to popular music such as “Yellow Submarine” by the Beatles. In this instance, Dr Ahern is extremely creative and a musician with an affinity for the Beatles. GenAI empowers instructors, even those lacking creative brilliance, to turn complex content into interactive and dynamic content such as games, clinical case scenarios, creative narratives, or even music and images. Thus, leveraging GenAI in a flipped classroom environment can reduce instructor workload while improving learning outcomes.

**Figure 5.**
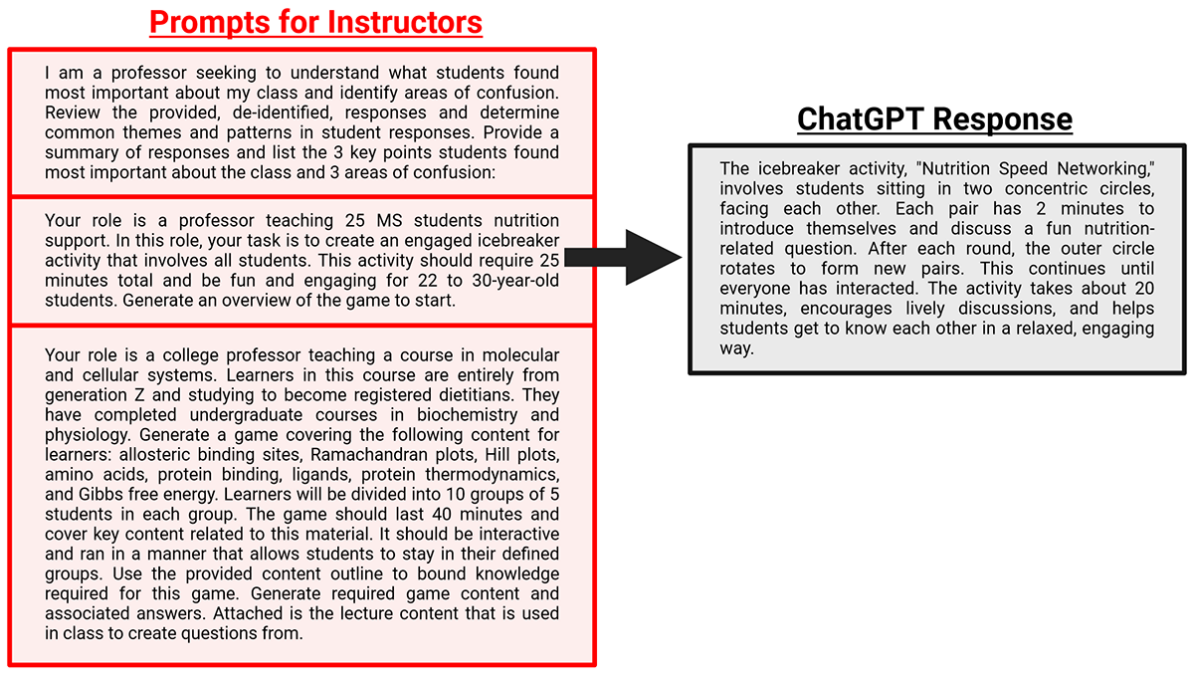
Example instructor prompts for leveraging generative artificial intelligence. Three example instructor input prompts are shown (red box) with representative ChatGPT (accessed on December 10, 2024) output (black box) for the middle prompt (black arrow). A key aspect for instructors is to clearly define the level of instruction being provided, type of learner being instructed, with narrowly defined content scope relative to learning objectives. The latter can be accomplished by inputting content outlines, lecture slides, or narrative summaries.

Gamification of course content is one mechanism toward merging GenAI tools with positive learner outcomes. GenAI can quickly generate games (eg, Kahoot!, bingo, Jeopardy!, crossword puzzles, or quiz show questions) using content outlines, slides, or even lecture as input (eg, Figure 5). Games are a low stress means of retrieval practice while promoting an interactive and engaged classroom experience [20]. GenAI can also generate complex clinical practice scenarios with rich hypothetical patient details. These scenarios require learners to use critical thinking and diverse knowledge outputs in a similar method to group-based socratic questioning used in medical education. One area we have had great success in using GenAI is developing gap analysis surveys for learners to assess knowledge levels upon course entry, midcourse for progression, and end of course for effectiveness relative to course objectives. GenAI can quickly generate gap assessments using course learning objectives, prerequisite course content outlines and turnkey instructor needs to provide immediate input on needed content modifications when introducing new content. This is an effective approach to identify knowledge gaps or needs that an instructor may assume are covered in prior courses, or were covered but not retained. Finally, GenAI can be leveraged to streamline workflows before, during, and after content delivery. This includes using templates to generate individualized self-assessment tools for learners, providing narrative feedback for learners on correct and incorrect responses, turning content outlines into focused assessments, integrating lecture modalities under core and redundant learning objectives, and statistical analysis on batched learner responses to identify learning gaps post-content delivery (Figures1  6) [21]. Thus, improving learner outcomes using GenAI tools shows significant promise even with clear limitations, discussed below, and rapidly evolving tools. A central theme for instructors when considering GenAI integration into preplanning, execution, and postanalysis is to balance promoting motivation with opportunities for knowledge retrieval. Course guidelines and instructor expectations must be very clearly defined as to acceptable GenAI use during a given course. This is important considering the current environment where broad institutional or district policies may be lacking, or nonexistent, and variability in what is acceptable between different instructors and courses. Effective communication and clear policies and procedures remain the most important means to avoid academic misconduct or malfeasance. Adapting retrieval strategies to accommodate different learning styles, while ensuring inclusive and personalized learning experiences, is important yet challenging to implement in practice. GenAI holds promise for instructors as these tools provide opportunities to reduce activation barriers (eg, time constraints) toward delivering more effective content and meaningful assessments.

**Figure 6.**
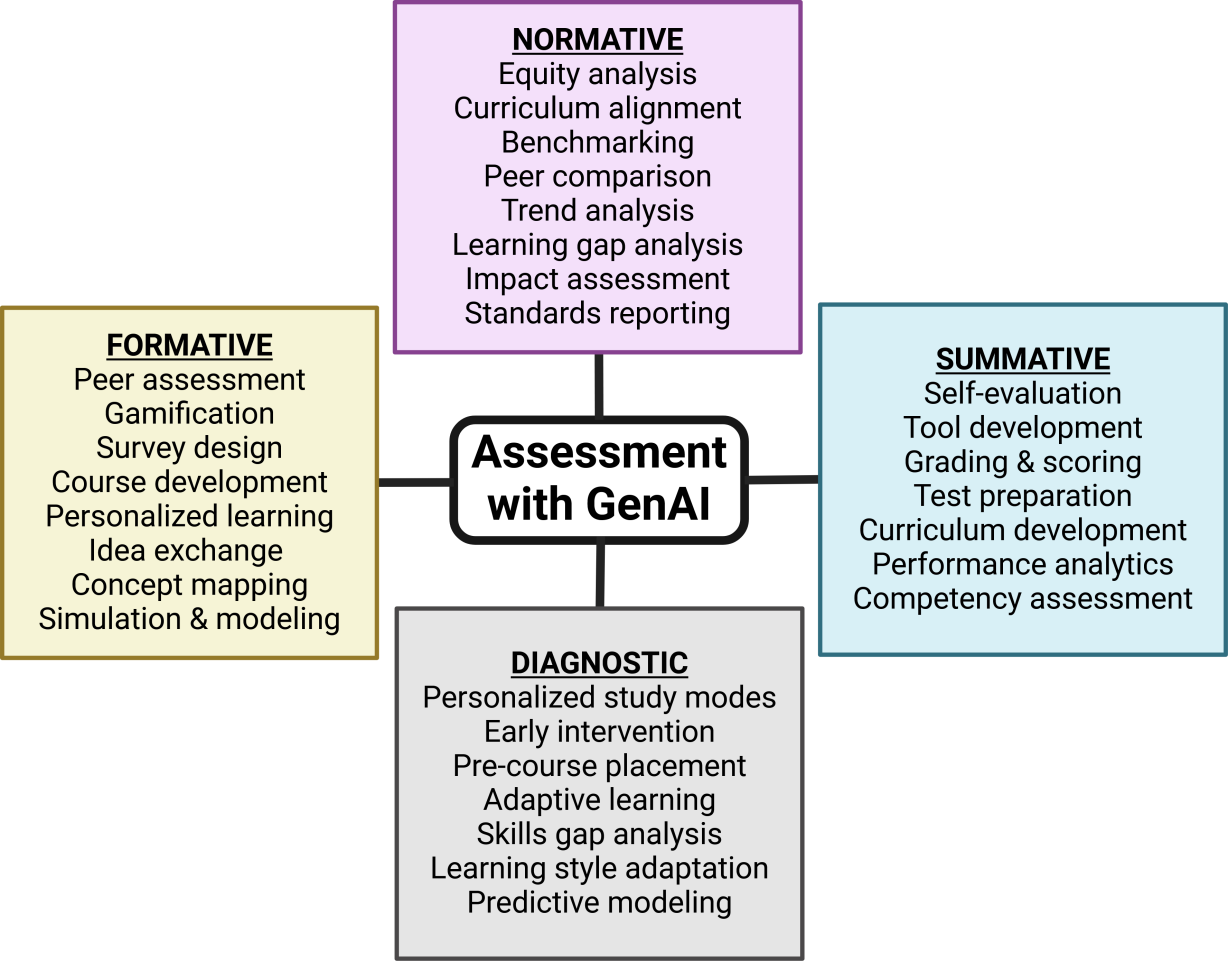
Generative artificial intelligence integration across assessment types. Generative artificial intelligence use across assessment modalities is shown. In formative assessment, generative artificial intelligence aids in creating interactive content and personalized feedback mechanisms. For summative assessment, generative artificial intelligence capabilities extend to evaluating overall learning achievements and generating comprehensive exams. Normative assessment with generative artificial intelligence focuses on establishing benchmarks and evaluating learning outcomes against standards, while diagnostic assessment leverages generative artificial intelligence for early detection of learning gaps and customization of learning experiences. The central position of “Assessment with GAI” emphasizes its role as a centralized tool, facilitating a comprehensive and integrated approach to educational assessment. GenAI: generative artificial intelligence.

## Current Limitations and Future Hurdles

Embracing GenAI tools holds promise for both learners and instructors, yet significant hurdles remain, and user caution is warranted in a rapidly evolving environment of GenAI capabilities, tools, ethics, and acceptable use policies and procedures. Accuracy of GenAI output is a critical aspect that requires careful consideration and diligence, especially when used as a training tool for future clinicians and scientists. LLMs are trained by large datasets and leverage analytics to produce predictions, not logic-derived integrations linking informed input to informed output [22]. Thus, a randomness can exist between prompt design and output obtained. Learners and instructors must both carefully assess and evaluate output from GenAI tools to detect “hallucinations” (ie, incorrect GenAI output that presents as correct). Outside of local application programming interface iterations, general releases of these GenAI models are trained on large datasets that may include unvalidated data from the internet. Thus, even if effort is made to include reliable and authoritative sources, these large training datasets may contain misinformation, biases, or outdated information from uncurated data. We have observed this on several occasions when implementing GenAI as an educational tool, where output sounds entirely factual and even referenced only to be completely incorrect with nonexistent references (this improved substantially in GPT-4ο and Claude-3.5 Sonnet, accessed on April 11, 2024). As of this submission, caution is still warranted even with significant improvements in model quality. One effective strategy to minimize accuracy issues is uploading content, such as lecture outlines or even slides, and designing focused prompts working from provided content. If possible, use an institutionally firewalled GenAI tool where content is not shared beyond immediate use. This works well to develop focused learner assessments. This leads to another limitation and hurdle: the rapid pace of GenAI tool development, improvement, and deployment has created an environment where time-limited instructors and digital native learners are increasingly overwhelmed with determining best practices, tools, methods, or even workflows. We have responded to this reality by developing open training courses (eg, on Canvas Learning Management System from Instructure) with frequent updates and ongoing informational seminars, often targeting instructors, to raise awareness of AI changes as they relate to clinical practice and pedagogy. While this rapidly evolving landscape of tools and capabilities is a challenge, it’s also an opportunity to leverage new features and expand impact.

In addition, a caveat to implementing “chatbot” style GenAI like ChatGPT in educational frameworks is the input prompt. The relationship between input prompt and output produced is so integrated that “prompt engineering” is a growing career emerging alongside GenAI [23]. Prompt engineering is the careful construction of input prompts or instructions for GenAI models to influence content, style, voice, depth, and even accuracy of resultant output. How input prompts are constructed is a primary determinant of what models produce, even down to small changes like omitting single words, changing adverbs, or using commas versus numbers for a list [24]. These small changes can produce vastly different results with biased, misleading, or inaccurate information [24]. Truly effective prompts are often complex and descriptive, striking a balance between specificity and openness [25]. Meaning, a prompt that is too specific may limit the ability to generate diverse or creative responses, and a prompt that is too open-ended may result in ambiguous or irrelevant output. The approach we use is constructing “Role+ Bounds+Inputs” style input prompts. In this formula, “Role” involves assigning the chatbot a specific job or identity for the analysis (eg, college professor teaching a specific course and learner type), “Bounds” will establish limitations and constraints for model operation (eg, academic context, subject matter, and level of expected answer), and “Inputs” includes relevant contextual information (eg, content outlines, rubrics, or slide summaries). Using a structured prompt guides GenAI models to produce more focused, accurate, and relevant responses. In addition, one can use a scaffolding approach with iterative prompts building toward a common theme or objective. The complexities of prompt engineering have been discussed elsewhere and leveraging the resources provides a deeper discussion of the benefits of a productive prompt while leveraging GenAI [26,27].

GenAI tools such as ChatGPT are proving transformative, impactful, and hold immense potential for enhancing the educational experience for both learners and instructors. Yet, this new reality is problematic considering the lack of transparency on training data content; ethical and socioeconomic implications; quality control and model accuracy; and the potential to perpetuate bias, loss of voice, and agency for both learners and instructors [21]. Learners should be empowered to make informed decisions regarding their participation in GenAI-related or driven activities, and instructors should encourage learners to ask questions, express concerns, and participate in shaping ethical guidelines related to GenAI use in curricula [11,21]. Furthermore, instructors and administrators have an expectation to proactively define clear policies and procedures for GenAI use in educational settings that provide flexibility for both learners and instructors. A major benefit to GenAI use in educational settings is its collaborative potential to rapidly generate personalized and dynamic content, yet this requires equity in understanding and use. Considering GenAI’s rapid evolution, this equity has always been absent in courses we have started since ChatGPT was released in November 2022. It’s incumbent on instructors to ensure learners are familiar with GenAI tools being used and available. Data-driven insights from GenAI analytics enable instructors to provide targeted support to individual learners as skills develop and courses progress. This can facilitate multimodal learning experiences, incorporating various media formats such as videos, audio, and interactive simulations mentioned above. Significant hurdles remain regarding GenAI use in higher education. These include data ownership and privacy, output accuracy, linking learner needs with instructor resources, and ensuring sufficient training to avoid equity and GenAI skills gaps when being used. Academic institutions are increasingly looking at internal, data-protected and firewalled, GenAI resources (eg, Microsoft CoPilot) yet there remain limited options for analyzing course content and metrics. Finally, ethical concerns associated with leveraging GenAI by both learners and instructors is a major (probably primary at most institutions) topic of discussion.

## Conclusions

Our experiences in leveraging GenAI across 5 academic semesters have, overall, been very positive. This implementation is from the perspective of informing and training both learners and instructors; establishing clear policies and procedures relating to academic misconduct at the department, college, and institutional levels; ensuring equity and ability in use; and constant vigilance regarding content accuracy and limitations. LLMs and GenAI have evolved through decades of iterative research across multiple disciplines including statistics, mathematics, and computer science [28]. They are not new technologies. Development of the transformer architecture in 2017 was a key transition point for the emergence of current “chatbots” gaining momentum in popular media and use [29]. This technology continues to evolve with diffusion, attention mechanism variants, and retrieval-enhanced transformer mechanisms being new examples of how GenAI technology is rapidly evolving [30]. Through leveraging large datasets with high-demand computational needs, current GenAI models show significant promise. The important point being, these models (eg, ChatGPT or Claude) excel at pattern recognition yet struggle with defining logical connections between training data and outputs produced (“reasoning”). This is an important caveat for application in higher educational settings focused on critical thinking, developing advanced knowledge and skills within specific disciplines, clinical training, and scientific discovery. As such, it’s essential for instructors, administrators, policymakers, institutions, districts, and learners to collaborate and communicate toward what this future will look like as GenAI models evolve. Through this collaboration, GenAI use in educational settings can be leveraged while minimizing negative aspects like potential misconduct, data privacy, algorithmic bias, accuracy, and equity concerns. We argue that GenAI can play a valuable role in higher education settings to improve learner motivation and knowledge retrieval while facilitating workflows and content generation for instructors. This viewpoint explores GenAI’s potential as an educational tool including alignment with learning theories (eg, behaviorism and cognitive load theory), implications for learners and instructors (eg, flipped classrooms and self-directed assessments), responsible implementation (eg, bias and equity), and evolving challenges (eg, hallucinations and misconduct).

## Abbreviations

AI: artificial intelligence
GAI: generative artificial intelligence
